# Associations between oxidative balance score and chronic kidney disease events in US adults: a population-based study

**DOI:** 10.1038/s41598-024-64147-9

**Published:** 2024-06-14

**Authors:** Yuewei Yin, Chenming Zhao, Yalin Niu, Jinchun Qi, Yanping Zhang, Baosai Lu

**Affiliations:** Department of Urology, The Second Hospital of Hebei Medical University, Shijiazhuang, 050000 China

**Keywords:** Oxidative balance scores, Chronic kidney disease, US adults, Cross-sectional study, National health and nutrition examination survey, Kidney diseases, Risk factors

## Abstract

Oxidative Balance Scores (OBS) are utilized to assess an individual's antioxidant status, encompassing both dietary and lifestyle factors that contribute to oxidative balance. This study investigates the relationship between OBS and chronic kidney disease (CKD) prevalence among U.S. adults, utilizing data from the National Health and Nutrition Examination Survey (NHANES) 2007–2018. The study involved a cross-sectional analysis of 13,373 individuals from NHANES, focusing on adults aged 20 years or older. OBS was calculated using 20 components, including dietary and lifestyle factors. CKD was identified based on albumin-to-creatinine ratio and estimated glomerular filtration rate, with patients stratified into mild, moderate, and high-risk groups. Statistical analysis included logistic regression models and restricted cubic splines to explore the OBS-CKD relationship. Our findings indicate a statistically significant negative correlation between OBS and CKD prevalence, particularly in mild and moderate-risk groups. Higher OBS quartiles were associated with a decreased likelihood of CKD (OR 0.70; 95% CI 0.53–0.92; P = 0.013). Restricted cubic splines indicated a non-linear, inverse association between OBS and CKD odds for the overall population (P for nonlinearity = 0.017). For mild and moderate CKD risk groups, the relationships were less pronounced (P for nonlinearity = 0.053 and 0.184, respectively), suggesting variability in the OBS-CKD link across different risk levels. The study highlights the potential of elevated OBS as a primary prevention measure for CKD, particularly in individuals with mild to moderate risk. These findings underscore the importance of antioxidant status in CKD risk management and encourage further research into the role of dietary and lifestyle factors in CKD prevention.

## Introduction

Chronic kidney disease (CKD), marked by progressive kidney function decline, is increasingly recognized as a significant global health challenge with substantial health and economic impacts^1,2^. In the United States, CKD prevalence in the general population is approaching 15%^3^, representing a considerable burden to both individuals and society. Consequently, identifying and managing modifiable risk factors for CKD has become a crucial public health priority. Factors such as lifestyle changes, dietary habits, smoking, and obesity, which are linked to oxidative stress, have contributed to the rising annual prevalence of kidney diseases^4–6^.

Oxidative stress is defined as a physiological imbalance where an overabundance of free radicals surpasses the body's inherent antioxidant defenses^7^. When the balance between prooxidants and antioxidants is disrupted, oxidative stress occurs, leading to damage in cells, tissues, and organs^8,9^. The kidney, owing to its high metabolic demands, is especially vulnerable to damage inflicted by oxidative stress^10^, a critical factor implicated in the advancement of renal diseases^7,11^. Oxidative Balance Scores (OBS) serve as a measure of an individual’s antioxidant status by considering both the antioxidant and pro-oxidant elements in their diet and lifestyle^12^, and have been correlated with biomarkers of inflammation and oxidative stress, such as F2-isoprostanes and C-reactive protein^13,14^. Recent research suggests that OBS measurement may be pivotal in detecting pro- and antioxidant exposure in individuals at risk for CKD^15^. While several studies have established a connection between OBS and CKD, they primarily focused on middle-aged and older populations. Therefore, we utilized data from the National Health and Nutrition Examination Survey (NHANES) 2007–2018 to conduct a comprehensive cross-sectional analysis, investigating the link between OBS and CKD prevalence among Americans, and further examining the relationship between OBS and different CKD risk stratification. Considering the influential roles of sodium and potassium in oxidative stress modulation and kidney function^16–18^, we included them as covariates in the examination of the relationship.

## Results

### Basic characteristics of the study population

Table 1 presents the basic characteristics of the study population across total and OBS quartile groups. Among 13,373 individuals, the mean age of all participants was 45.99 ± 17.23 years, with 6906 (weighted frequency, 50.64%) men and 6467 (49.36%) women. Of these participants, 69.76% were non-Hispanic white, 72.30% had more than a high school education, 55.05% were married, 6.68% had diabetes, and 25.35% had hypertension. Notably, eGFR decreased with increasing OBS quartiles (P < 0.001), while the ACR showed no significant differences across quartiles (P = 0.243). The prevalence of CKD was significantly higher in higher OBS quartiles (P < 0.001). The weighted overall prevalence of CKD in this study was 9.86%.

**Table 1:** Characteristics of the study sample (NHANES 2007–2018), weighted.

	Overall (N = 13,373)	Oxidative balance score				P-value
		Q1 (N = 1853)	Q2 (N = 4438)	Q3 (N = 4373)	Q4 (N = 2709)	
Age, years	44.71 ± 16.47	42.62 ± 14.91	44.76 ± 16.07	45.6 ± 16.98	44.75 ± 17.43	< 0.001
Sex, n (%)						
Men	6906 (50.64%)	1424 (77.36%)	2634 (58.76%)	1919 (39.87%)	929 (30.87%)	< 0.001
Women	6467 (49.36%)	429 (22.63%)	1804 (41.24%)	2454 (60.13%)	1780 (69.13%)	
RACE, n (%)						
Mexican American	1725 (7.26%)	267 (8.14%)	572 (6.83%)	538 (6.95%)	348 (8.00%)	< 0.001
Other Hispanic	1259 (5.24%)	166 (5.83%)	367 (4.21%)	440 (5.71%)	286 (6.07%)	
Non-Hispanic White	5949 (69.76%)	901 (71.27%)	2138 (73.56%)	1880 (68.13%)	1030 (63.54%)	
Non-Hispanic Black	2641 (9.52%)	322 (8.32%)	749 (7.38%)	884 (10.06%)	686 (13.99%)	
Other Race	1799 (8.22%)	197 (6.44%)	612 (8.02%)	631 (9.15%)	359 (8.41%)	
Education, n (%)						< 0.001
Less than high school education attainment	1932 (8.67%)	239 (8.00%)	559 (7.25%)	649 (9.05%)	485 (11.52%)	
High school graduate	2674 (19.03%)	354 (17.87%)	802 (17.01%)	882 (18.95%)	636 (24.32%)	
Has more than a high school education	8767 (72.30%)	1260 (74.13%)	3077 (75.74%)	2842 (72.00%)	1588 (64.16%)	
Marital status, n (%)						< 0.001
Married	6928 (55.05%)	425 (55.62%)	1068 (58.78%)	1206 (54.45%)	822 (47.86%)	
Never married	2924 (21.97%)	972 (23.43%)	2461 (21.42%)	2247 (20.42%)	1248 (24.71%)	
Other status	3521 (22.98%)	456 (20.95%)	909 (19.79%)	920 (25.13%)	639 (27.43%)	
HDL, mg/dL	55.10 ± 16.87	51.64 ± 15.63	54.81 ± 16.46	56.50 ± 17.00	56.02 ± 17.98	< 0.001
TC, mg/dL	193.90 ± 41.30	191.80 ± 41.23	194.00 ± 41.13	194.20 ± 40.16	195.00 ± 43.67	0.225
Diabetes, n (%)						0.056
Yes	1247 (6.68%)	143 (6.35%)	384 (6.04%)	408 (6.71%)	312 (8.22%)	
No	12,126 (93.32%)	1710 (93.65%)	4054 (93.96%)	3965 (93.79%)	2397 (91.78%)	
Hypertension, n (%)						0.753
Yes	3908 (25.35%)	480 (25.13%)	1256 (25.01%)	1304 (25.16%)	868 (26.59%)	
No	9465 (74.65%)	1373 (74.87%)	3182 (74.99%)	3069 (74.84%)	1841 (73.41%)	
Serum potassium, mmol/L	3.98 ± 0.33	4.03 ± 0.31	4.00 ± 0.32	3.97 ± 0.32	3.93 ± 0.34	< 0.001
Serum sodium, mmol/L	139.30 ± 2.26	139.30 ± 2.24	139.30 ± 2.27	139.40 ± 2.21	139.30 ± 2.34	0.279
eGFR, mL/min 1.73 m^2^	97.78 ± 19.41	99.75 ± 17.62	97.96 ± 18.55	97.07 ± 20.05	97.07 ± 21.24	< 0.001
ACR, mg/g	22.38 ± 175.30	17.04 ± 92.26	21.98 ± 186.60	21.85 ± 120.20	28.65 ± 265.20	0.243
CKD, n (%)						< 0.001
Yes	1639 (9.86%)	167 (7.40%)	501 (9.06%)	535 (9.83%)	436 (13.62%)	
No	11,734 (90.14%)	1686 (92.60%)	3937 (90.94%)	3838 (90.87%)	2273 (86.38%)	

Mean ± standard error for continuous variables, percentages (%) for categorical variables.

Q1, 0–10 units; Q2, 10–20 units; Q3, 20–30 units; Q4, 30–40 units.

*HDL* high-density lipoprotein, *TC* total cholesterol, *eGFR* estimates of glomerular filtration rate, *ACR* albumin-to-creatinine ratio, *CKD* chronic kidney disease.

### The association between oxidative balance score and chronic kidney disease

**Figure 1:**
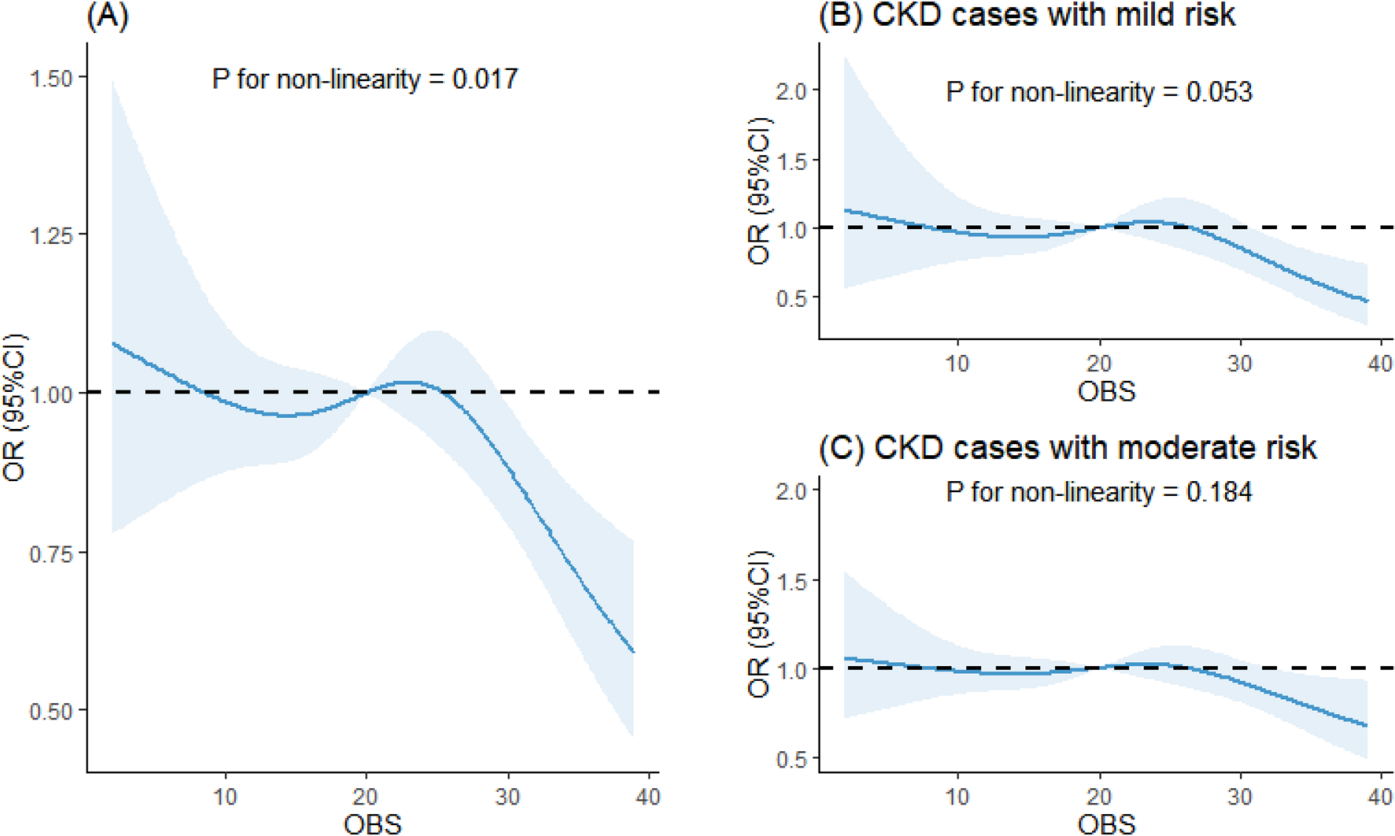
The dose–response relationships between oxidative balance score and chronic kidney disease. (**A**) RCS curves describing the dose–response relationship between oxidative balance score and CKD; (**B**) RCS curves describing the dose–response relationship between oxidative balance score and mild–risk stratification for CKD; (**C**) RCS curves describing the dose–response relationship between oxidative balance score and moderate–risk stratification for CKD. The solid lines and shaded areas represent the central risk estimates and 95% CIs. The following covariates were adjusted for: age, sex, race, marital status, education, HDL, TC, diabetes, hypertension, serum sodium and serum potassium.

Table 2 shows that the increases in OBS are associated with a lower odds of CKD (P for trend < 0.001). Compared with those in the OBS quartile 1, individuals in the 4th quartile of the OBS had decreased odds of CKD (Model 1: OR 0.51; 95% CI 0.39–0.66; P < 0.001). This association remained significant, after adjusting for BMI, HDL, TC, serum potassium, serum sodium, diabetes, hypertension, and demographic variables (Model 2: OR 0.71; 95% CI 0.54–0.94; P = 0.016; Model 3: OR 0.70; 95% CI 0.53–0.92; P = 0.013). Supplementary Table S2 demonstrates the OBS's relationship with eGFR and albuminuria. A higher OBS was associated with better eGFR scores and less albuminuria in weighted multivariable linear regression analysis. In the highest OBS quartile, there was a significant association with improved eGFR (Model 3: Estimate = − 1.01, P < 0.001) and increased albuminuria (Model 3: Estimate = 11.07, P = 0.064). Restricted cubic splines revealed a non-linear, negative relationship between OBS and the odds of CKD within all samples (P for nonlinearity = 0.017; Fig. 1A).

**Table 2:** Multivariable logistic regression analysis of the association between oxidative balance score and chronic kidney disease, weighted.

	Actual population numbers	Weighted population	Model 1		Model 2		Model 3	
			OR (95% CI)	P-value	OR (95% CI)	P-value	OR (95% CI)	P-value
Q1	1853	16,620,874	Ref		Ref		Ref	
Q2	4438	41,482,417	0.80 (0.61,1.06)	0.120	0.93 (0.70,1.23)	0.599	0.90 (0.67,1.21)	0.468
Q3	4373	36,064,965	**0.73 (0.58,0.93)**	**0.011**	0.99 (0.76,1.30)	0.959	0.96 (0.73,1.26)	0.740
Q4	2709	19,897,440	**0.51 (0.39,0.66)**	**< 0.001**	**0.71 (0.54,0.94)**	**0.016**	**0.70 (0.53,0.92)**	**0.013**
P for trend			–		**< 0.001**		**< 0.001**	

Bolded denotes significant.

Q1, 0–10 units; Q2, 10–20 units; Q3, 20–30 units; Q4, 30–40 units.

Model 1: adjusted for no covariates.

Model 2: adjusted for age, sex, race, education and marital status.

Model 3: Model 2 + HDL + TC + serum potassium + serum sodium + diabetes + hypertension.

Table 3 presents the associations between OBS and different CKD risk stratifications based on weighted logistic regression. The significant relationship between OBS and CKD was found in the mild-risk (P for trend < 0.001) and moderate-risk groups (P for trend = 0.001), but not in the high-risk group (P for trend = 0.240). In the mild-risk group, participants in the 3rd (Model 1: OR 0.57; 95% CI 0.37–0.89; P = 0.014) and 4th quartiles (Model 1: OR 0.36; 95% CI 0.22–0.57; P < 0.001) of the OBS had lower odds of CKD than those in the first quartile. The above associations of the mild-risk group were not found in the fully adjusted model; however, after adjusting for all potential covariates, OBS Q4 (Model 3: OR 0.70; 95% CI 0.51–0.96; P = 0.029) were significantly associated with a lower likelihood of CKD in the moderate-risk group. Further, Supplementary Table S3 shows significant links between OBS and eGFR within the mild and moderate risk groups, absent in the high-risk group, with the 4th OBS quartile demonstrating consistently reduced CKD odds (Model 3: Estimate = − 1.68 and Estimate = − 1.35; P < 0.01). Additionally, elevated OBS quartiles corresponded with a notable increase in albuminuria within the mild and moderate risk groups (Model 3: Estimate = 2.51 and 3.95, respectively; P < 0.001) (Supplementary Table S4). Figure 1B,C depict the linear relationship of OBS with mild (P for nonlinearity = 0.053) and moderate (P for nonlinearity = 0.184) risk stratifications of CKD.

**Table 3:** Multivariable logistic regression analysis of the association between oxidative balance score and chronic kidney disease risk stratification, weighted.

Stratification factors	Cases/non-cases	Model 1		Model 2		Model 3	
		OR (95% CI)	P-value	OR (95% CI)	P-value	OR (95% CI)	P-value
CKD cases with mild risk	461/11,734						
Q1		Ref					
Q2		0.73 (0.44,1.23)	0.239	1.09 (0.63,1.9)	0.755	1.05 (0.59,1.88)	0.863
Q3		**0.57 (0.37,0.89)**	**0.014**	1.12 (0.68,1.85)	0.649	1.05 (0.63,1.76)	0.843
Q4		**0.36 (0.22,0.57)**	**< 0.001**	0.70 (0.41,1.19)	0.185	0.66 (0.38,1.13)	0.131
P for trend				**< 0.001**		**< 0.001**	
CKD cases with moderate risk	1001/11,734						
Q1		Ref					
Q2		0.77 (0.55,1.07)	0.117	0.84 (0.61,1.16)	0.292	0.81 (0.58,1.12)	0.197
Q3		0.81 (0.61,1.07)	0.137	1.01 (0.74,1.37)	0.966	0.96 (0.71,1.31)	0.811
Q4		**0.54 (0.39,0.73)**	**< 0.001**	**0.71 (0.52,0.96)**	**0.027**	**0.70 (0.51,0.96)**	**0.029**
P for trend				**0.002**		**0.002**	
CKD cases with high risk	177/11,734						
Q1		Ref					
Q2		1.32 (0.64,2.74)	0.446	1.44 (0.67,3.12)	0.346	1.48 (0.70,3.14)	0.304
Q3		0.76 (0.33,1.74)	0.515	0.94 (0.39,2.27)	0.892	0.87 (0.36,2.13)	0.764
Q4		0.94 (0.41,2.17)	0.888	1.28 (0.50,3.26)	0.61	1.31 (0.52,3.30)	0.557
P for trend				0.240		0.240	

Bolded denotes significant.

Q1, 0–10 units; Q2, 10–20 units; Q3, 20–30 units; Q4, 30–40 units.

Model 1: adjusted for no covariates.

Model 2: adjusted for age, sex, race, education and marital status.

Model 3: Model 2 + HDL + TC + serum potassium + serum sodium + diabetes + hypertension.

### Subgroup analysis

**Figure 2:**
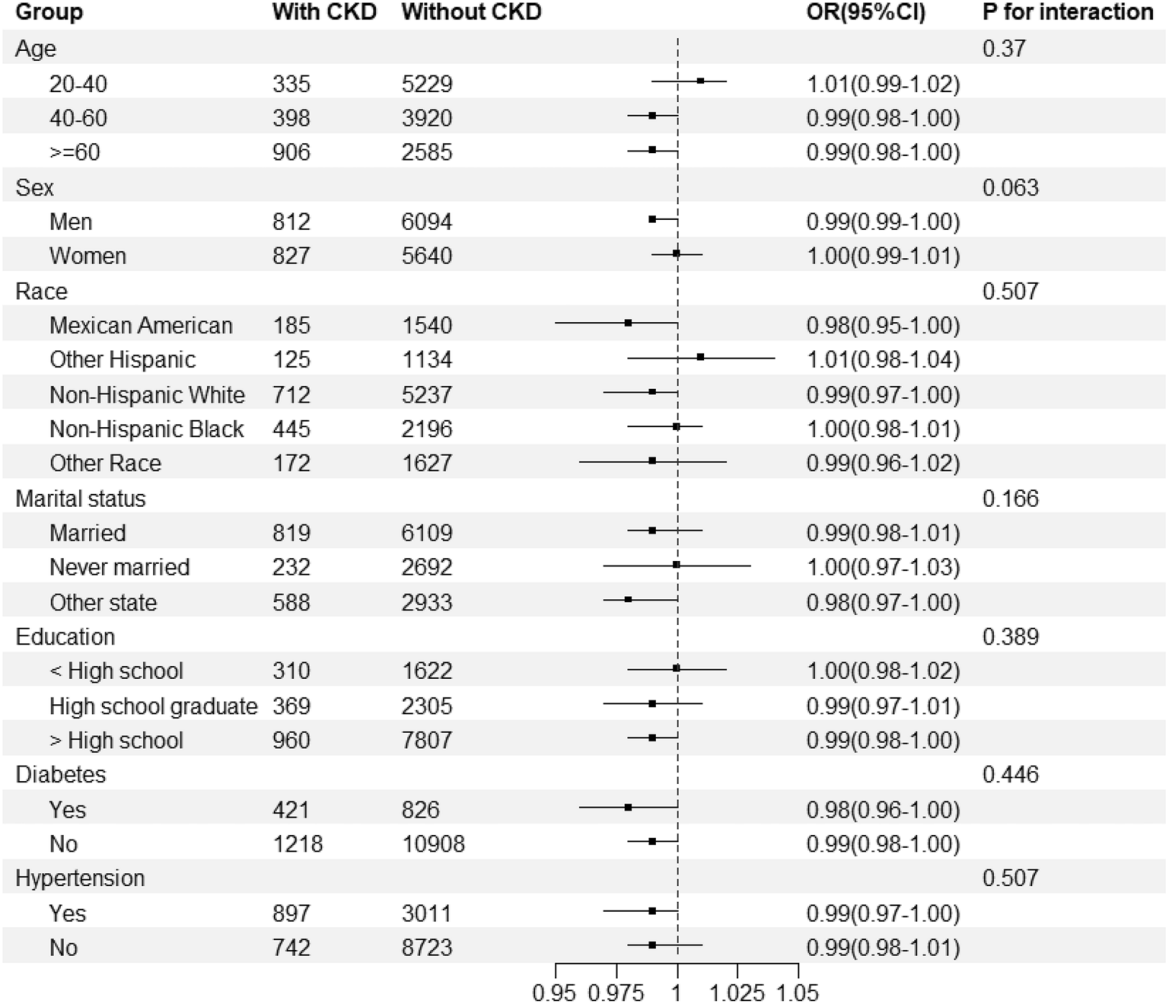
Forest plot for subgroup analysis. Subgroup analysis was stratified by age, sex, race, marital status, education, diabetes, and hypertension.

Figure 2 shows the results of subgroup analyses and the interactions between CKD and categorical variables in the study. The results showed a consistent negative correlation between OBS and CKD in individuals with varying disease statuses and demographic factors (P-values > 0.05 for all interactions).

## Discussion

In our analysis of 13,373 participants from the NHANES 2007–2018, we explored the relationship between OBS and CKD prevalence among US adults. The study revealed that individuals in the highest OBS quartile (Q4) had a lower likelihood of prevalent CKD compared to those in the lowest quartile (Q1), after adjusting for all relevant covariates. Notably, this association was statistically significant in the mild and moderate CKD risk groups, but it was not observed in the high-risk group. Analysis using restricted cubic splines indicated a negative non-linear correlation between OBS and CKD prevalence. These findings suggest that elevated OBS may be beneficial in mitigating the risk of developing CKD, with a more pronounced effect observed in individuals with mild to moderate risk of the disease.

Our results are consistent with previous studies that have demonstrated an inverse connection between OBS and the prevalence of CKD. OBS, a multidimensional measure integrating various diet and lifestyle elements, acts as a barometer for an individual's antioxidant status^12^. In line with our results, Ilori et al. discovered a notable inverse link between higher OBS quartiles and CKD risk, though they did not find a significant association with albuminuria or the development of end-stage renal disease (ESRD)^15^. Similarly, a recent Korean study confirmed this trend, showing that increased OBS significantly lowers the risk of developing CKD in Asian populations^19^. Moreover, studies indicate a rise in oxidative markers in both atherosclerotic lesions and the circulating plasma of CKD patients, pointing to a pro-oxidant state in CKD^20,21^. These collective findings highlight the critical role of oxidative balance in both the onset and advancement of CKD. Interestingly, our study observed this relationship predominantly in individuals with mild to moderate CKD risk, rather than in those with high risk. This indicates the potential of a healthy diet and lifestyle, which mitigate oxidative stress, in curtailing the risk of new-onset CKD.

Although the etiology of CKD is complex, oxidative stress emerges as a pivotal factor in its pathogenesis. Oxidative stress is a consequence of the imbalance between the production of reactive oxygen species (ROS) and the antioxidant capacity of the cell^22^. The pathogenesis of CKD is significantly influenced by an imbalance between excessive ROS production and weakened antioxidant defense systems^7,23^. This imbalance originates from the activation of various enzyme systems, including nicotinamide adenine dinucleotide phosphate (NADPH) oxidase, lipoxygenase, xanthine oxidase, uncoupled nitric oxide synthase (NOS), and the mitochondrial respiratory chain. At the same time, there's a reduction in the effectiveness of crucial antioxidants such as superoxide dismutase (SOD), catalase, selenium-based glutathione peroxidase, and paraoxonases (PON). This disparity, marked by increased pro-oxidant activities and reduced antioxidant capabilities, leads to the oxidation of vital macromolecules, resulting in tissue damage and functional decline in CKD^24^. Furthermore, the overproduction of ROS plays a direct role in the onset and progression of CKD, contributing to associated conditions like proteinuria, arterial hypertension, and diabetes mellitus^23–26^. Notably, oxidative stress manifests in the early stages of CKD, with increased ROS levels contributing to the progressive decline in GFR and vascular complications^27,28^ Thus, oxidative stress plays a key role in the pathophysiology of CKD development.

This study has several notable strengths. First, we utilize data from the NHANES database, ensuring the reliability of our findings through the application of appropriate weights and adjustments for confounding variables during statistical analysis. A key methodological strength is the use of the OBS, a comprehensive score combining dietary and lifestyle factors, to evaluate individuals' antioxidant status. We also conducted risk stratification for CKD cases to investigate the impact of OBS across different CKD risk categories. Nevertheless, the study is not without limitations. Being a cross-sectional analysis, it can only establish an association, not a causative link, between OBS and CKD. Furthermore, the utilization of OBS is constrained to contexts in which specific data are available, necessitating additional research to clarify its direct applicability in clinical practice. Another notable limitation is the potential for selection bias, attributed to the exclusion of numerous participants due to incomplete data. Additionally, our reliance on dietary information from the initial 24-h dietary recall interview for estimating dietary components introduces the possibility of bias. Moreover, the study does not incorporate endogenous measurements of antioxidants and oxidative stress, which may offer further insights into the OBS–CKD relationship.

In conclusion, our research found a negative correlation between OBS and the prevalence of CKD in the American adult population. Notably, this correlation was statistically significant within groups at mild and moderate risk of CKD, rather than high-risk. The analysis revealed a non-linear inverse trend between increasing OBS and the prevalence of CKD. Consequently, elevated OBS levels may serve as a primary preventative strategy against CKD, potentially contributing to a reduction in its overall prevalence.

## Methods

### Study population

The NHANES is a nationally representative, cross-sectional survey conducted by the National Center for Health Statistics (NCHS) of the Centers for Disease Control and Prevention (CDC). This ongoing program with a 2-year reporting cycle targets the non-institutionalized civilian population of the United States and employs a complex multistage probability sampling design. Data collection in NHANES is carried out through a two-step process, starting with in-home interviews followed by physical examinations and laboratory tests at a Mobile Examination Center (MEC). Ethical approval for the survey was granted by the NCHS Research Ethics Review Board, and all participants provided written informed consent. Publicly accessible details on the NHANES' data collection methods and methodology are available on the official website (cdc.gov/nchs/nhanes.htm).

**Figure 3:**
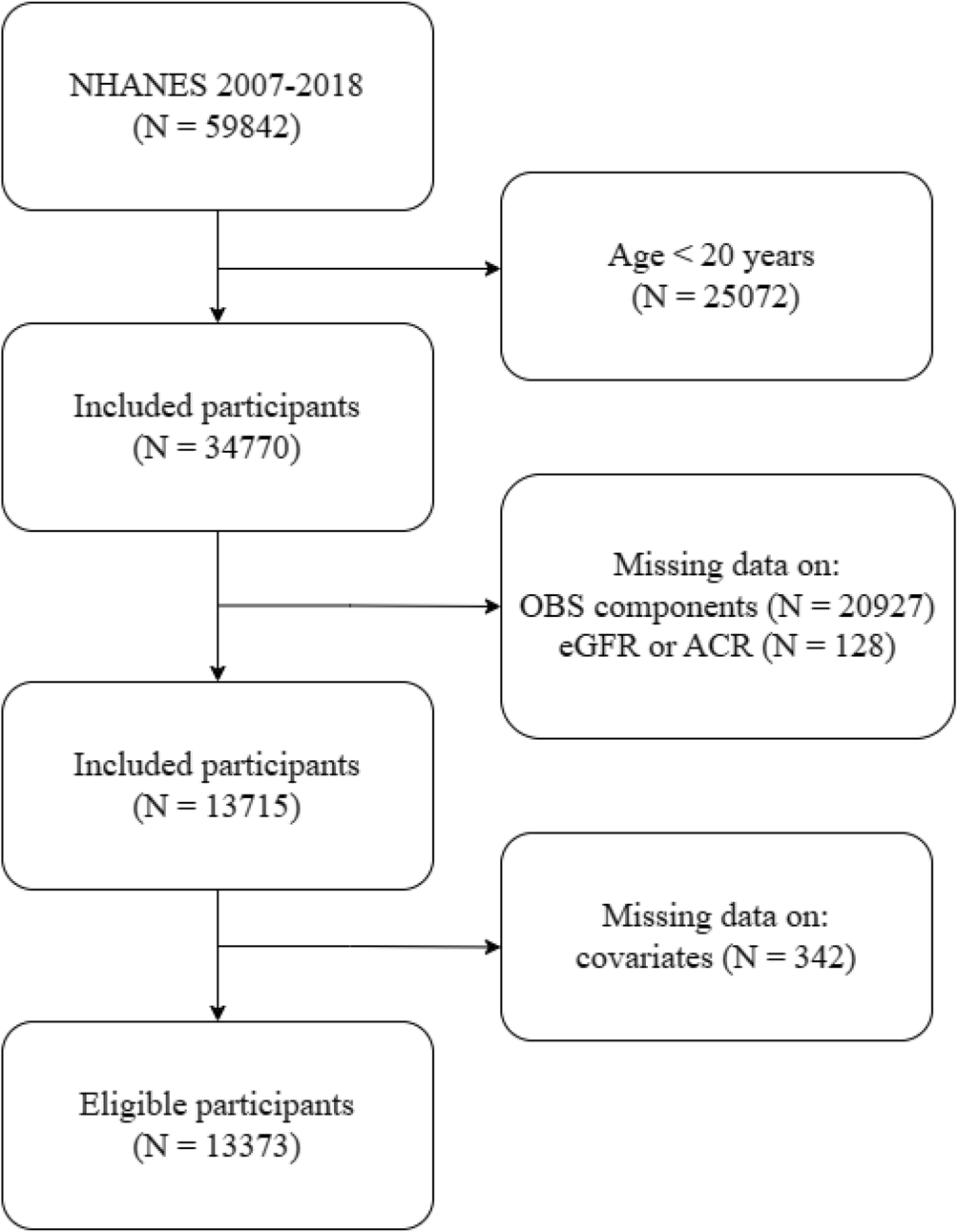
Flow chart of participant selection based on NHANES database from 2007 to 2018.

For this study, data from the six cycles (2007–2008, 2009–2010, 2011–2012, 2013–2014, 2015–2016, and 2017–2018) were combined, resulting in an initial sample size of 59,842 individuals. Subsequently, the analysis was focused on 34,770 adults aged 20 years or older. Among them, 20,927 participants with incomplete data on OBS components were removed. We further excluding 128 participants missing key measurements that estimated glomerular filtration rate (eGFR) or albumin-to-creatinine ratio (ACR). Additionally, we eliminated data with incomplete covariates (i.e., age, sex, race/ethnicity, education, marital status, diabetes, hypertension, high-density lipoprotein (HDL), total cholesterol (TC), serum potassium, and serum sodium) (n = 342). Ultimately, the final sample for this study comprised 13,373 individuals. A detailed flowchart illustrating the participant selection process is presented in Fig. 3.

### Oxidative balance scores

OBS were calculated by incorporating both dietary and lifestyle factors, using the method described in the related literatures^29,30^. This comprehensive approach involved selecting twenty components associated with oxidative stress—16 dietary and 4 lifestyle elements (Supplementary Table S1). These components were grouped as follows: (1) dietary antioxidants including fiber, β-carotene, riboflavin, niacin, vitamin B6, total folate, vitamin B12, vitamin C, vitamin E, and minerals like calcium, magnesium, zinc, copper, and selenium, (2) dietary pro-oxidants comprising total fat and iron, (3) lifestyle antioxidants represented by physical activity, and (4) lifestyle pro-oxidants such as alcohol consumption, smoking habits, and body mass index (BMI). The dietary component of OBS was derived from 24-h dietary recall interviews conducted in the NHANES Mobile Examination Center, without accounting for nutrients from dietary supplements or medications. Dietary components were categorized into tertiles, with dietary antioxidants scoring 0–2 points ascendingly and dietary pro-oxidants scoring reversely from 2–0 points. For lifestyle components, physical activity was classified as low (< 150 min/week), moderate (150–300 min/week), or high (> 300 min/week), with corresponding scores of 0, 1, and 2 points, based on the 2018 Physical Activity Guidelines for Americans^31^. Alcohol consumption was scored 0–2 points based on sex-specific consumption patterns, while smoking was evaluated using serum cotinine levels and assigned 0–2 points inversely across tertiles. BMI was calculated as weight in kilograms divided by the square of height in meters and categorized into normal (< 25 kg/m^2^), overweight (25–29.9 kg/m^2^), or obese (≥ 30 kg/m^2^), with scores of 2, 1, and 0 points, respectively. Overall, the OBS ranged from 0 to 40 points, with the total score being the sum of points from all sixteen dietary and four lifestyle components.

### Chronic kidney disease

In this study, we defined the dependent variable as the presence of chronic kidney disease (CKD). CKD was primarily identified by two criteria: a urinary albumin-to-creatinine ratio (ACR) exceeding 30 mg/g or an estimated glomerular filtration rate (eGFR) falling below 60 mL/min/1.73 m^2^^32^. 2021 CKD Epidemiology Collaboration equation is used to calculate eGFR based on serum creatinine levels^33^. The measurement of serum creatinine is employed the enzymatic method. Urine albumin levels were determined using a solid-phase fluorescence immunoassay, and urine creatinine was quantified through the modified Jaffé kinetic method. Additionally, we conducted a risk stratification of CKD patients based on the staging of CKD and the grading of albuminuria. These categories were as follows: mild risk, encompassing individuals with normal to mildly increased albuminuria (ACR < 30 mg/g); moderate risk, including those with moderately increased albuminuria (ACR: 30–300 mg/g); and high risk, comprising individuals with severely increased albuminuria (ACR > 300 mg/g)^34^.

### Covariates

We further incorporated various factors potentially linked to OBS and the prevalence of CKD, drawing on existing clinical knowledge and research. These factors included sociodemographic variables, medical conditions, and biomedical data. All of sociodemographic variables were gathered from the NHANES questionnaire. Race was classified into Mexican American, Other Hispanic, Non-Hispanic White, Non-Hispanic Black, and Other Race. Educational attainment was divided into three levels: less than high school, high school graduate (including those with a high school diploma or an equivalency diploma like the General Educational Development), and more than high school education. Marital status was categorized as married, not married, and other (including separated, widowed, or divorced). Biomedical data and medical conditions taken into consideration included HDL (mg/dL), TC (mg/dL), diabetes (yes or no), hypertension (yes or no), serum potassium (mmol/L), and serum sodium (mmol/L). Body mass index (BMI) was calculated by dividing weight in kilograms by the square of height in meters. The presence of hypertension and diabetes was determined based on self-reported diagnoses. Both HDL and TC levels were measured using an enzymatic method with the Roche Modular P chemistry analyzer at the University of Minnesota. Detailed information about these measurement procedures is available in the NHANES Procedure Manuals.

### Statistical analysis

Our study used sample weights in all analyses to ensure representativeness, taking into account the complex multi-stage probabilistic sampling design of NHANES. Categorical variables were presented as unweighted counts and weighted percentages, whereas continuous variables were expressed as weighted means with standard errors. Weighted analysis of variance (ANOVA) and weighted chi-square tests for continuous and categorical variables, respectively. The OBS was categorized into quartiles (Q1, Q2, Q3, Q4), with Q1 serving as the reference group. Logistic regression models were utilized to explore the association between OBS and CKD prevalence. These models varied in terms of adjustment for confounding factors: Model 1 was unadjusted, Model 2 adjusted for sociodemographic variables (age, sex, ethnicity, marital status, and education level), and Model 3 further adjusted for potential correlates including age, sex, ethnicity, marital status, education level, high-density lipoprotein (HDL), total cholesterol (TC), diabetes, hypertension, serum potassium, and serum sodium. Our analysis also extended to the examination of OBS's association with eGFR and albuminuria, applying identical modeling approaches. Additionally, CKD patients were classified into mild, moderate, and high-risk categories. Logistic regression models were again utilized to evaluate the relationship between OBS and different CKD risk stratifications, using the lowest quartile of OBS (Q1) as the reference group. We explored dose–response dynamics between OBS and CKD prevalence using restricted cubic spline curves, with model adjustments mirroring those in Model 3.

Subgroup analyses were stratified according to age, sex, race, marital status, education, hypertension, and diabetes to assess the consistency of the OBS–CKD association across different demographic and clinical profiles. We assessed interactions among covariates using likelihood ratio tests, adjusting models for relevant covariates excluding the stratification variables.

All statistical analyses were conducted using R software, version 4.2.1 (https://www.r-project.org/). For all tests, a two-sided P-value of less than 0.05 was considered to indicate statistical significance.

### Supplementary Information

Supplementary Tables.

## Data Availability

The data underlying this article will be shared on reasonable request to the corresponding author.
