# Supplementary material for: Associations between oxidative balance score and chronic kidney disease events in US adults: a population-based study
Source: Sci Rep. 2024 Jun 14;14:13743. doi: 10.1038/s41598-024-64147-9 (PMC11178767; doi:10.1038/s41598-024-64147-9)
Supplement: Supplementary file 1 — Supplementary Tables. [file 41598_2024_64147_MOESM1_ESM.pdf]

Associations between Oxidative Balance Score and Chronic Kidney Disease Events in US Adults: A Population-Based Study

Authors: Yuewei Yin[1†], Chenming Zhao[1†], Yalin Niu[1], Jinchun Qi[1], Yanping Zhang[1], Baosai Lu[1]*

Supplementary Tables

Table S1. Detailed scoring criteria for oxidative balance score components.
Table S2. Multivariable linear regression analysis of the association of oxidative balance score with eGFR and albuminuria, weighted.
Table S3. Multivariable logistic regression analysis of the association between oxidative balance score and eGFR across different chronic kidney disease risk stratifications, weighted.
Table S4. Multivariable logistic regression analysis of the association between oxidative balance score and albuminuria across different chronic kidney disease risk stratifications, weighted.

Table S1. Detailed scoring criteria for oxidative balance score components.

Component	Scoring Detail
**Dietary antioxidant**	
Fiber	0 points for low tertile, 1 point for middle tertile, 2 points for high tertile
β-carotene	
Riboflavin	
Niacin	
Vitamin B6	
Total folate	
Vitamin B12	
Vitamin C	
Vitamin E	
Calcium	
Magnesium	
Zinc	
Copper	
Selenium	
**Dietary pro-oxidant**	
Total fat	2 points for low tertile, 1 point for middle tertile, 0 points for high tertile
Iron	
**Lifestyle antioxidant**	
Physical activity	0 points for <150 min/week, 1 point for 150–300 min/week, 2 points for >300 min/week
**Lifestyle pro-oxidant**	
Alcohol consumption	0 points for high intake, 1 point for moderate intake, 2 points for low/no intake
Smoking	2 points for non-smokers, 1 point for former smokers, 0 points for current smokers
BMI	2 points for <25 kg/m^2; 1 point for 25–29.9 kg/m^2, 0 points for ≥30 kg/m^2

Table S2. Multivariable linear regression analysis of the association of oxidative balance score with eGFR and albuminuria, weighted.

	Actual population numbers	Weighted population	Model 1		Model 2		Model 3	
			Estimate (95% CI)	P-value	Estimate (95% CI)	P-value	Estimate (95% CI)	P-value
**eGFR**								
Q1	1853	16,620,874	Ref		Ref		Ref	
Q2	4438	41,482,417	**-1.79 (-2.96, -0.61)**	**0.003**	-0.61 (-1.59, 0.38)	0.225	-0.71 (-1.72, 0.30)	0.166
Q3	4373	36,064,965	**-2.68 (-3.90, -1.46)**	**<0.001**	**-1.28 (-2.32, -0.24)**	**0.016**	**-1.37 (-2.41, -0.33)**	**0.011**
Q4	2709	19,897,440	**-2.68 (-4.12, -1.23)**	**<0.001**	**-1.99 (-3.12, -0.86)**	**<0.001**	**0.70 (-3.26, -1.01)**	**<0.001**
**Albuminuria**								
Q1	1853	16,620,874	Ref		Ref		Ref	
Q2	4438	41,482,417	3.97 (-1.55, 9.49)	0.156	3.63 (-1.93, 9.19)	0.198	4.63 (-0.76, 10.02)	0.091
Q3	4373	36,064,965	5.37 (-0.74, 11.48)	0.084	3.27 (-3.17, 9.72)	0.315	4.53 (-1.84, 10.89)	0.161
Q4	2709	19,897,440	**15.22 (3.74, 26.70)**	**0.009**	11.56 (-0.16, 23.27)	0.053	11.07 (-0.67, 22.82)	0.064

* Bolded denotes significant.

Q1, 0-10 units; Q2, 10-20 units; Q3, 20-30 units; Q4, 30-40 units.

Model 1: adjusted for no covariates.

Model 2: adjusted for age, sex, race, education and marital status.

Model 3: Model 2 + HDL + TC + serum potassium + serum sodium + diabetes + hypertension.

Table S3. Multivariable logistic regression analysis of the association between oxidative balance score and eGFR across different chronic kidney disease risk stratifications, weighted.

Stratification factors	Cases/non-cases	Model 1		Model 2		Model 3	
		Estimate (95% CI)	P-value	Estimate (95% CI)	P-value	Estimate (95% CI)	P-value
CKD cases with mild risk	461/11734						
Q1				Ref			
Q2		**-1.69 (-2.91, -0.47)**	**0.007**	-0.34 (-1.37, 0.68)	0.508	-0.44 (-1.48, 0.61)	0.409
Q3		**-2.25 (-3.55, -0.95)**	**<0.001**	-1.00 (-2.07, 0.07)	0.067	-1.05 (-2.11, 0.01)	0.053
Q4		**-2.16 (-3.74, -0.58)**	**0.008**	**-1.56 (-2.67, -0.44)**	**0.007**	**-1.68 (-2.80, -0.57)**	**0.004**
CKD cases with moderate risk	1001/11734						
Q1				Ref			
Q2		**-1.43 (-2.56, -0.30)**	**0.014**	-0.60 (-1.50, 0.29)	0.186	-0.65 (-1.56, 0.26)	0.158
Q3		**-1.94 (-3.20, -0.69)**	**0.003**	**-1.17 (-2.17, -0.16)**	**0.024**	**-1.17(-2.16, -0.17)**	**0.022**
Q4		-1.03 (-2.32, 0.27)	0.119	**-1.30 (-2.30, -0.30)**	**0.012**	**-1.35 (-2.35, -0.35)**	**0.009**

CKD cases with high risk	177/11734						
Q1				Ref			
Q2		**-1.52 (-2.69, -0.35)**	**0.011**	-0.48 (-1.47, 0.50)	0.330	-0.58 (-1.56, 0.41)	0.247
Q3		**-1.80 (-3.09, -0.51)**	**0.007**	**-1.09 (-2.13, -0.04)**	**0.042**	**-1.11 (-2.16, -0.07)**	**0.036**
Q4		-1.00 (-2.41, 0.41)	0.163	**-1.24 (-2.35, -0.13)**	**0.028**	**-1.32 (-2.43, -0.22)**	**0.020**

* Bolded denotes significant.
Q1, 0-10 units; Q2, 10-20 units; Q3, 20-30 units; Q4, 30-40 units.
Model 1: adjusted for no covariates.
Model 2: adjusted for age, sex, race, education and marital status.
Model 3: Model 2 + HDL + TC + serum potassium + serum sodium + diabetes + hypertension.

Table S4. Multivariable logistic regression analysis of the association between oxidative balance score and albuminuria across different chronic kidney disease risk stratifications, weighted.

Stratification factors	Cases/non-cases	Model 1		Model 2		Model 3	
		Estimate (95% CI)	P-value	Estimate (95% CI)	P-value	Estimate (95% CI)	P-value
CKD cases with mild risk	461/11734						
Q1				Ref			
Q2		0.16 (-0.43, 0.74)	0.596	0.42 (-0.16, 1.01)	0.154	0.47 (0.59,1.88)	0.124
Q3		**1.05 (0.35, 1.76)**	**0.00389**	**1.37 (0.67, 2.07)**	**<0.001**	**1.33 (0.63,1.76)**	**<0.001**
Q4		**2.42 (1.49, 3.34)**	**<0.001**	**2.61 (1.69, 3.52)**	**<0.001**	**2.51 (0.38,1.13)**	**<0.001**
CKD cases with moderate risk	1001/11734						
Q1				Ref			
Q2		**1.76 (0.03, 3.50)**	**0.046**	**1.90 (0.26, 3.5)**	**0.024**	**2.15 (0.50, 3.80)**	**0.011**
Q3		**2.25 (0.21, 4.28)**	**0.031**	**2.08 (0.16, 4.00)**	**0.034**	**2.27 (0.41, 4.14)**	**0.017**
Q4		**4.72 (2.95, 6.50)**	**<0.001**	**4.16 (2.42, 5.90)**	**<0.001**	**3.95 (2.16, 5.73)**	**<0.001**

CKD cases with high risk	177/11734						
Q1				Ref			
Q2		2.80 (-2.74, 8.35)	0.318	2.44 (-3.15, 8.03)	0.388	3.47 (-2.01, 8.94)	0.211
Q3		4.52 (-1.77, 10.81)	0.157	2.52 (-4.19, 9.23)	0.458	4.38 (-2.33, 11.08)	0.198
Q4		**14.89 (1.92, 27.86)**	**0.025**	11.36 (-1.71, 24.43)	0.088	11.74 (-1.58, 25.06)	0.083

* Bolded denotes significant.
Q1, 0-10 units; Q2, 10-20 units; Q3, 20-30 units; Q4, 30-40 units.
Model 1: adjusted for no covariates.
Model 2: adjusted for age, sex, race, education and marital status.
Model 3: Model 2 + HDL + TC + serum potassium + serum sodium + diabetes + hypertension.
